# Study protocol and preliminary baseline characteristics of a VA multi-site, mixed methods, randomized controlled study evaluating supported employment provided to veterans with opioid use disorder

**DOI:** 10.1186/s12991-024-00533-x

**Published:** 2024-12-18

**Authors:** Mercy Mumba, Teresa Granger, George Mugoya, Stephen Brackett, Junfei Lu, Emily Lund, Charles Lynn, Anchal Ghera, Whitney Gay, Lori Davis

**Affiliations:** 1 Center for Substance Use Research and Related Condition, Capstone College of Nursing, The University of Alabama, 650 University Blvd East, Tuscaloosa, AL 35401 USA; 2 Department of Educational Studies in Psychology, Research Methodology and Counseling, The University of Alabama, 520 Colonial Drive, Tuscaloosa, AL 35401 USA; 3 Birmingham VA Health Care System, 700 19th St South, Birmingham, AL 35233 USA; 4 Tuscaloosa VA Medical Center, 3701 Loop Rd, Tuscaloosa, AL 35404 USA

**Keywords:** Individual placement and support, Opioid use disorders, Vocational rehabilitation, Mental health, MOUD

## Abstract

Opioid Use Disorder (OUD) is a pervasive and devastating public health crisis that continues to take a heavy toll on individuals and communities across the United States. In 2021, approximately 473,000 veterans misused opioids in the past year. In the context of their military service and post-service life, Veterans with OUD often encounter unique barriers to recovery, including the reintegration into civilian society and the pursuit of stable, meaningful employment. The path to recovery from OUD is inextricably linked to the restoration of a stable and purposeful life, a fact underscored by the interplay of substance use, mental health, and employment outcomes. These factors necessitate a comprehensive approach to treatment that extends beyond mere pharmacological interventions. One such approach is Individual Placement and Support (IPS), a well-established evidence-based practice that focuses on supporting individuals with severe mental illness in their pursuit of competitive employment. The primary objective of this manuscript is to describe a two-arm, multi-site RCT designed to rigorously evaluate the efficacy of IPS when provided to veterans with OUD and provide the baseline demographics and characteristics of the participants who have enrolled to date. The central hypothesis guiding this research is that IPS can significantly improve vocational, psychosocial, and treatment outcomes of veterans in recovery from OUD, ultimately leading to a more successful reintegration into civilian life. Our study is timely as the VA has expanded IPS services to veterans with SUD this past year. Thus, this study is one of the first to examine IPS in a subpopulation of veterans with a SUD and may provide actionable data to support sustainment of IPS with this population.

## Introduction

Opioid Use Disorder (OUD) is a pervasive and devastating public health crisis that continues to take a heavy toll on individuals and communities across the United States. In 2017, the US. Department of Health and Human Services (HHS) declared the opioid crisis a public health emergency [1]. Over 9.2 million people in the US. misuse opioids [2]. The impact of this crisis is acutely felt within the veteran population, where the prevalence of OUD and its associated complications remains a formidable challenge. In 2021, approximately 473,000 veterans misused opioids in the past year [2]. In the context of their military service and post-service life, veterans with OUD often encounter unique barriers to recovery [3], including the reintegration into civilian society and the pursuit of stable, meaningful employment.

The path to recovery from OUD is inextricably linked to the restoration of a stable and purposeful life, a fact underscored by the interplay of substance use, mental health, and employment outcomes [4–6]. These factors necessitate a comprehensive approach to treatment that extends beyond pharmacological interventions. One such approach is Individual Placement and Support (IPS) [7], a well-established evidence-based practice that focuses on supporting individuals with severe mental illness in their pursuit of competitive employment [8, 9].

IPS emphasizes the principles of rapid job placement, personalized job development, ongoing job support, and a “place-then-train” philosophy [10]. IPS is a manualized approach with a validated treatment fidelity scale [11]. While IPS has demonstrated its effectiveness in improving employment outcomes among individuals with severe mental illness [8, 9], and among veterans with PTSD [12, 13] and broad-spectrum psychiatric disabilities [14], its applicability and efficacy in the unique context of veterans with OUD remain largely unexplored. The confluence of substance use and employment challenges makes this population a particularly challenging and, at the same time, vital target for intervention research.

Harrison and colleagues conducted a systematic review of IPS for persons with substance use disorders (SUD) [15]. Their results yielded five randomized controlled trials (RCTs) and two studies that were non-randomized controlled trials [16–20]. Of the RCTs, only one included a specific sample with OUD as opposed to other types of SUD [16]. The other six studies evaluated IPS among samples with co-occurring mental illness and SUD [17–20]. Those with OUD specifically were not reported or separated in analyses from other SUDs. Nonetheless, across all of the RCTs, IPS participants had better employment outcomes than control groups. Of note, two of the RCTs were with a veteran sample [18, 20]. Among the two non-randomized IPS studies, participants obtained employment at higher rates of employment than the general rate of employment for individuals with co-occurring SUD within the state the study was conducted in [21]. Results of the non-randomized study showed that IPS participants with and without SUD obtained competitive employment at similar rates [22].

Lones and colleagues evaluated the efficacy of IPS for 45 patients with OUD enrolled in a methadone treatment program [16]. This is the only study found to evaluate IPS among an OUD population. Participants were randomized to either receive a 6-month IPS intervention or to a waitlist. During the 6-month intervention period, half of the IPS group attained competitive employment compared to only 5% among the waitlist control, which increased to 22% after 12-months but stayed at 50% for the IPS group.

The primary objective of this manuscript is to describe a two-arm, multi-site RCT designed to rigorously evaluate the efficacy of IPS when provided to veterans with OUD and provide the baseline demographics and characteristics of the participants who have enrolled to date. The central hypothesis guiding this research is that IPS can significantly improve vocational, psychosocial, and treatment outcomes of veterans in recovery from OUD, ultimately leading to a more successful reintegration into civilian life. Our study is timely as the VA has expanded IPS services to veterans with SUD this past year. Thus, this study is one of the first to examine IPS in a subpopulation of veterans with a SUD and may provide actionable data to support sustainment of IPS with this population. Our study could improve our understanding of vocational rehabilitation needs, employment challenges and solutions, and relapse prevention for veterans with OUD.

## Methods

### Ethics approval and consent

This study is approved by the Institutional Review Boards of the Tuscaloosa VA Medical Center, The Birmingham VA Health Care System, and The University of Alabamain accordance with the Declaration of Helsinki. Written informed consent is obtained prior to participating in any study procedures. The study is monitored by an independent Data and Safety Monitoring Board and has a Certificate of Confidentiality approved by the National Institutes of Health.

### Overview of design, aims, and hypotheses

The purpose of this study is to evaluate the efficacy of IPS provided to veterans with OUD using a mixed methods approach. Our first aim is to conduct a prospective, multi-site, RCT to determine the efficacy of IPS compared to treatment-as-usual non-IPS vocational rehabilitation (non-IPS-VR). Consenting and eligible veterans who are currently unemployed or under-employed and are in treatment for OUD are randomized to either IPS or non-IPS-VR. Each participant receives IPS or non-IPS-VR and have outcomes assessed for 18 months. We hypothesize that participants who receive IPS will have significantly more weeks worked in a competitive job over 18 months compared to non-IPS-VR services. We also hypothesize that compared to non-IPS-VR, IPS recipients will earn significantly more income from competitive jobs and be significantly more likely to achieve steady employment, defined as working in a competitive job for 50% of the 18-month period. Our second aim is to explore and identify contextual barriers and facilitators of implementing rehabilitation services in both study arms and to achieving competitive employment among veterans with OUD. We will evaluate the implementation and employment challenges, optimal functioning, and OUD treatment adherence. We are employing a community-based participatory research (CBPR) approach to achieve this aim. True to CBPR approach, the interview guide for the present study was developed through an iterative interview process with various stakeholders, including employers of individuals with OUD, IPS and vocational rehabilitation specialists, Veterans, and researchers with expertise in this field. These various stakeholders were asked to identify various contextual barriers and facilitators of employment for Veterans with OUD. From these interviews, various overarching themes were developed to guide the development of the interview guide to further explore Veterans perspectives in the current study. The fundings of the barriers and facilitators will be published in a separate manuscript. For this study, we are also utilizing an iterative process of qualitative individual interviews with veterans with OUD that were randomized to IPS or non-IPS-VR. Our final aim is to evaluate the impact of IPS compared to non-IPS-VR on adherence to medications for OUD (MOUD) and rates of OUD relapse. We hypothesize that compared to non-IPS-VR, veterans who receive IPS will be significantly more likely to adhere to MOUD (i.e., those on MOUD only) and to have lower rates of OUD relapse.

### Eligibility criteria

Eligible participants are (1) consenting US. military veterans who are ≥ 19 years of age; (2) currently unemployed or underemployed (i.e., working < 20 h/week in competitive employment, or currently employed in a job that is not meaningful or well-matched to the veteran); (3) desiring to work in a competitive job; and (4) have a history of current or lifetime OUD according to DSM-5. Veterans with a history of traumatic brain injury may be included as long as they do not have a severe cognitive disorder. The key to attaining and sustaining employment in the context of a recovery model is being recognized for the potential contributions one has made in the past and can once again address moving forward. Eligible veterans are either unemployed, marginally employed, or caught in the trap of working in entry level jobs unrelated to their past education, training and/or military experience. The absence of work or career choices with relevance or meaning to the veteran’s life can be a determinant of accepting and sustaining treatment adherence. Meaningful work is often described as employment in a valued environment or performing work tasks related to one’s personal preferences or life experiences, military or non-military.

Exclusion criteria include (1) a current diagnosis of schizophrenia, schizoaffective disorder or other psychotic disorders; (2) diagnosis of dementia or severe cognitive disorder; (3) presence of current severe and unstable medical condition or terminal illness, that would contraindicate study participation or expose them to an undue risk; (4) unlikely that participant can complete the study, e.g., expected deployment, incarceration, long-term hospitalization, or relocation from the vicinity; (5) active suicidal or homicidal ideation making it unsafe for the participant to be included; or (6) current participation in another vocational interventional trial.

### Recruitment procedures

Participants are recruited from the Tuscaloosa VA Medical Center and the Birmingham VA Health Care System as they naturalistically present for treatment in vocational rehabilitation services, residential treatment program, outpatient mental health clinics, and addiction recovery programs, are referred to the study, respond to the IRB-approved advertisement/letter, or make personal inquiry into the study. During the 3-year enrollment period, we plan for at least 180 veterans to be directly offered consent, 140 to sign consent, 120 to be randomized, and 100 veterans to complete the study at the two sites. Participants are modestly compensated for each baseline and the bimonthly assessment visits.

### Randomization and follow-up schedule

Participants who meet eligibility criteria are randomized (1:1) to either IPS or non-IPS-VR. Randomization uses a permuted block design of randomly varying block sizes stratified by site. Once assignment is made, the participant is analyzed in that group regardless of future events or services rendered, in accordance with the intent-to-treat principle.

Once informed consent is obtained, baseline assessments are administered. Participants are assessed at baseline and every 2 months (months 2, 4, 6, 8, 10, 12, 14, 16) and then final assessment at month 18 for employment outcomes and secondary outcomes. At each research visit, participants are assessed for the occurrence of adverse events or serious adverse events. The 18-month follow-up period allows for start-up time involved in each intervention prior to establishing competitive work and allows enough time for a participant to establish steady competitive work and achieve the secondary outcome goals.

### Clinical assessments

At baseline, the Clinical Research Coordinator (CRC) collects baseline demographics and characteristics, including age, gender, race, ethnicity, marital status, education level, military history (i.e., branch, period of service, combat exposure), status of housing and transportation, status of VA and non-VA disability, psychiatric treatment history, past and current treatment for OUD, and work history, including length of unemployment, type of previous job(s), and longest duration of competitive work. The Mini-International Neuropsychiatric Interview for DSM-5 is conducted by an investigator or CRC to confirm OUD diagnosis and document comorbidities [23]. Repeated measures include: the Patient Health Questionnaire-9 (PHQ-9) [24]; the Generalized Anxiety Disorder Scale (GAD-7) [25]; Brief Resilience Scale (BRS) [26]; Perceived stress Scale-10 (PSS-10) [27]; Quality of Life Scale (QOLS) [28]; Community Assessment Inventory (CAI) [29]; Alcohol Abstinence Self-Efficacy (modified for OUD) [30]; the Tobacco, Alcohol, Prescription Medications, and Other Substance (TAPS) Part-2 Tool [31]; and the Columbia Suicide Severity Rating Scale (C-SSRS) [32].

### Employment outcome assessments

The primary outcome is the number of weeks worked during the 18-months following randomization. A “week worked” is defined as working either full- or part-time in a competitive job during a 7-day period. Competitive employment is defined as a job that pays at least minimum wage based on wages, salary, or commission, is in a setting not set aside for those with mental illness or disabilities, and is not transitional work. The exploratory employment outcome will be ‘steady worker’ status, defined as obtaining competitive employment for at least 50% of the active follow-up period (i.e. ≥ 32 weeks). Longitudinal employment data will include number of weeks, days, and hours worked in a competitive job, dollars earned, type of job(s), number of jobs, and job satisfaction. Job satisfaction is measured with the Indiana Job Satisfaction Scale (IJSS) [33]. Employment outcomes and IPS or non-IPS-VR services received will be collected using a calendar approach and verified by copies of pay documents (e.g., pay stub, direct deposit notices, pay checks).

### MOUD outcome assessment

We are allowing Veterans on all types of MOUD (e.g., methadone, suboxone, naltrexone etc.) to be part of the study. We will ensure that type of MOUD will be controlled for in all statistical models at the end of the study. Adherence and relapse (i.e., opioid misuse ≥ 4 times/month) will be measured using the TAPS-2 Tool and pharmacy records. Adherence is defined as the number of days MOUD was received as indicated during the last 4 weeks of the intervention period.

### Study setting

This study will be conducted within the vocational rehabilitation services at Tuscaloosa VA Medical Center and the Birmingham VA Health Care System.

### Individual placement and support intervention

IPS is a manualized model of supported employment that focuses on obtaining and maintaining competitive employment that align with the participants’ skills, abilities, knowledge, career interests, and prevocational training [34]. The IPS model encompasses eight principles: (1) *Competitive Employment:* The IPS intervention assists participants to directly engage in a competitive job rather than prevocational training or set-aside sheltered jobs. (*2) Systematic Job Development:* IPS specialists build an employer network based on participant’s interests, developing relationships with local employers by making systematic contacts. (3) *Rapid Job Search:* IPS specialists use a rapid job search approach to help participants obtain employment as soon as possible, rather than providing lengthy pre-employment assessment, training, and counseling. (4) *Integrated Services:* The IPS intervention is closely integrated with the treatment team, in this case the outpatient substance use treatment or MOUD program. (5) *Benefits Counseling:* IPS specialists help veterans obtain personalized, understandable, and accurate information about their VA, Social Security, Medicaid, and other government entitlements. (6) *Zero Exclusion:* IPS embraces the notion of “zero exclusion” criteria, i.e., does not preclude participants because of level of disability, work history, or active substance use. (7) *Time-Unlimited Support*: IPS follow-along vocational supports are continued for as long as the participant wants and needs the support (in this case for 18-months due to the time limits of the study). The unlimited support provides help during job transitions if the first job is not the preferred or best-matched job. (8) *Worker Preferences:* The IPS intervention is based on the participant's preferences and choices rather than the IPS specialist’s or treatment team’s judgements. The caseload size for an IPS Specialist is 20–25 clients. The IPS specialists provides all phases of employment services such as intake, assessment, job development, and job coaching.

### Treatment-as-usual non-*IPS* vocational rehabilitation services

The participants assigned to non-IPS-VR will participate in available VA VR services at the study sites, except for IPS. The non-IPS-VR services may include two types of Compensated Work Therapy (CWT): Transitional Work or Community Based Employment Services. Transitional Work refers to a set-aside, minimum-wage, short-term job, typically in the VA setting. Community-Based Employment Services involves a community job search, placement in a competitive job, with limited follow-along support that typically ends after the veteran begins a new job.

### *IPS* specialist training and fidelity monitoring

The IPS specialists initially attend a 5-week online IPS course provided by the IPS Employment Center [35]. The IPS manual entitled *Supported Employment: Applying the Individual Placement and Support (IPS) Model to Help Clients Compete in the Workforce* [34] and the VHA VR Program Guide are provided to the IPS specialists for training and reference. The IPS trainer/mentor provides technical assistance via weekly teleconferences and intermittent on-site visits throughout the study timeline.

On an annual basis, the IPS fidelity monitor will make an on-site visit and conduct a 2-day fidelity monitoring. Fidelity monitoring involves observing the IPS specialists in the field during job development and interaction with participants, interviews participants, interviews with clinical treatment providers, interviews leadership, and review of the VA electronic medical record. Using the 25-item Supported Employment (SE) Fidelity Scale, the IPS fidelity monitor provides feedback on the fidelity rating to the investigators to aid in working with the IPS specialists to ensure high quality IPS services. The IPS fidelity monitor also evaluates the non-IPS-VR treatment arm using the same methods to ensure that the control arm is rated ≤ 55 on the SE Fidelity Scale (i.e., not supported employment). If non-IPS-VR is rated > 55 on the SE Fidelity Scale (i.e., non-IPS-VR takes on similarities to IPS), the research team will not interfere or modify usual care and will subsequently explore this variable in the analysis. The SE Fidelity Scale is comprehensive, detailed, and research-based [36, 37]. The relationship between IPS fidelity and employment outcomes using the 25-item IPS fidelity scale has been shown to have predictive validity [38].

### Data analyses

Descriptive statistics will summarize the socio-demographic and clinical characteristics of the sample at baseline. Continuous variables will be summarized by means, standard deviations, medians, and ranges. Categorical variables will be summarized by frequencies and rates. Although we expect randomization to produce equivalent groups, we will compare the two groups (IPS vs. non-IPS-VR) on baseline variables, using t-tests for the continuous variables and chi-square tests for categorical variables.

### Primary outcome analysis

The target sample size is 120 randomized participants (60 per study arm). The sample size was based on our most recent IPS study at the Tuscaloosa VA Medical Center that evaluated IPS in the primary care setting as nearly half of the participants had a SUD diagnosis. We expect that more than 84% will complete the intervention as randomized and greater than 95% will provide analyzable employment data. The between-group effect size was 0.55 in favor of IPS in the prior study, for which our projected sample size (54 per group with > 2 months data) would provide > 81% power. Very conservatively assuming that only 90% provide analyzable data this yields approximately 54 participants per group. This provides 80% power to detect a difference in means between groups of 0.54 standard deviations for a continuous outcome. The primary employment outcome is the number of weeks with competitive employment over the 18-month follow-up period. A week scored as “worked” is one in which a competitive job was held for any number of hours or days during that week. A week scored as “not worked” is one in which there was no competitive job held, or there were no employment data (i.e., early exit or missing data). The analysis of the primary outcome will use longitudinal mixed-effects regression models considering the cumulative number of weeks worked at each assessment as the dependent variable and study arm (IPS vs. non-IPS-VR) as the main independent variable adjusted for gender. Study site will be treated as a random effect, as will participant to account for the nesting of participants within sites and the multiple observations per person. We will initially specify the covariance structure of the repeated outcomes as unstructured and will proceed respectively to autoregressive and exchangeable structures if convergence issues are encountered. Prior to final modeling we will examine longitudinal plots of the employment data by group to evaluate whether the most appropriate model and any possible interaction effect appears to be additive (linear regression) or multiplicative (suggesting negative binomial regression), whether time should be included as categorical or linear, and whether it appears that a group*time interaction term is required to faithfully model the data.

The primary hypothesis that participants randomized to IPS will work more weeks over the 18-months compared to those randomized to non-IPS-VR will be evaluated using predicted marginal means (i.e., LS-means) to estimate the difference in hours worked and test significance at the *p* < 0.05 level. Adhering to the principle of intent-to-treat, participants may discontinue the treatment intervention, but will be encouraged to remain in the study for outcome assessments for the 18-month follow-up period and will be analyzed as-randomized.

The exploratory employment outcome is the dichotomous classification of whether each participant is a ‘steady worker’ operationalized as a participant who holds a competitive job for at least 50% of the 18-month follow-up period. The analysis for this outcome will follow the same structure as for the primary employment outcome but using logistic rather than linear regression. Similar analyses for overall intervention effects will also consider outcomes of total time worked (days and hours), income earned from competitive sources, and the type and number of jobs held. These analyses will be descriptive and will be used to identify the overall time-trends of employment between the groups, including when the highest probability of employment is reached and whether the efficacy appears to decline after a period of time. This information can be used to further refine future IPS interventions to maximize the durability of intervention effects.

### Secondary outcomes analysis

The effect of treatment on each of the psychological clinical measures will be analyzed using a longitudinal mixed-effects regression model similar to the methods described above to model the longitudinal trends in employment outcomes. The dependent variable will be the outcome score at baseline and each follow-up time point. Each follow-up visit will be categorized by the weeks since enrollment and an indicator for calendar month when the visit occurred will also be included in the model to control for seasonality. We will formally test for differences in outcomes at 18 months using predicted-marginal means. All hypothesis tests are pre-specified so there is no compelling reason to adjust the alpha level for multiple comparisons, and because they are likely to be correlated, a Bonferroni correction would be expected to be overly conservative. However, to aid interpretation we will use the Benjamini–Hochberg method to control the family-wise false-discovery rate for the secondary outcomes to be ≤ 10%.

Both between-treatment condition and within-treatment condition effect sizes for primary and secondary outcomes will be presented: Cohen’s *d* for continuous outcomes and the number needed to treat for dichotomous outcomes. An estimate of the 95% confidence interval will accompany each effect size in order to guide interpretation. Effect sizes indicate the magnitude of the treatment effect based on clinical significance and are independent of sample size and hence statistical significance.

### Qualitative data analyses

With regard to the qualitative aim to examine barriers and facilitators of treatment implementation and employment outcomes via a 90-min semi-structured interview, we will recruit participants from both study arms that reaches a sample size sufficient for data saturation to occur through a thematic analysis [39]. Transcribed interview data will be reviewed by three study team members experienced in qualitative data analysis procedures. These independent analysts will use an inductive process to explore themes to identify the contextual implementation barriers and facilitators, followed by the creation of subthemes as guided by the data. If analysts identify what appear to be new themes during the coding process, a deductive approach will be used to determine if these newly identified themes inform the existing themes (i.e., act as a sub-theme) or extend the main themes. We will use a combined inductive-deductive approach to coding that is based in the situational context of the data [40].

### MOUD analyses

An additional aim is to compare IPS to non-IPS-VR in terms of adherence to MOUD and relapse into heavy opiate use. We expect that MOUD adherence can be treated as a continuous variable in regression models. However, prior to modeling we will examine its distribution, to determine if this is appropriate. Relapse will be treated in two ways, as a binary indicator if any relapse as determined by TAPS (defined as opioid misuse ≥ 4 times/mo.), and the total number of self-reported days of misuse. The first dichotomous outcome will require logistic regression. The second approach will depend on the observed distribution and may be treated as either negative binomial or continuous. We will also explore the differences between groups in rates of relapse. The exploratory analysis will construct longitudinal models similar to those in our first aim considering outcomes of weekly adherence and relapse with that week’s employment status as well as lagged employment status (e.g., employment in previous weeks) as covariates to more directly examine the possible role of employment outcomes in explaining adherence and/or relapse. Further analyses may also include psychosocial covariates.

## Results

Since January 2023, 41 participants have been enrolled and randomized with 25 participants randomized to IPS (10 to site one, 15 to site two) and 16 to non-IPS-VR (3 to site one, 13 to site two). Enrollment will remain open until we achieve our enrollment threshold, no later than August 2025. Tables 1, 2 and 3 describe the baseline and clinical characteristics of the sample to date. To provide a contextual understanding of the study sample, we provide two study vignettes describing two cases, one from each study arm, as shown in Vignettes #1 and #2. Since enrollment and follow-up are still ongoing, no outcomes are presented in this report.

**Table 1:** Baseline Demographics and Military History

Variable	IPS (*N* = 25)		Non-IPS-VR (*N* = 16)		Total (*N* = 41)	
	*M*	*SD*	*M*	*SD*	*M*	*SD*
Age (years)	44.4	10	54	11.26	48.2	11.4
Prior-Year Annual Income ($US)	$34,480	$25,587	$16,383	$14,990	$16,383	$14,990
	Frequency	Percent	Frequency	Percent	Frequency	Percent
Sex						
Male	21	84.0	14	87.5	35	85.4
Female	4	16.0	2	12.5	6	14.6
Race						
White	14	56.0	6	37.5	20	48.8
Black or African American	8	32.0	9	56.3	17	41.5
Asian, Mixed, or Other	3	12.0	1	6.3	4	9.8
Ethnicity						
Not Hispanic or Latino	25	100	16	100	41	100
Marital Status						
Single	7	28.0	4	25.0	11	26.8
Married	3	12.0	2	12.5	5	12.2
Divorced	13	52.0	7	43.8	20	48.8
Other	2	8.0	3	18.8	5	12.2
Education						
Less than High School Diploma	1	4.0	–	–	1	2.4
High School Diploma/GED	8	32.0	3	18.8	11	26.8
Vocational Training	11	44.0	11	68.8	22	53.7
Associate or Bachelor’s Degree	5	20.0	2	12.5	7	17.1
Branch of Service						
Army	11	44.0	10	62.5	21	51.2
Navy	9	36.0	2	12.5	11	26.8
Air Force	3	12.0	3	18.8	6	14.6
Other	2	8.0	1	6.3	3	7.3
Period of Service						
Vietnam Conflict	–	–	1	6.3	1	2.4
MAY-1975 to JUL-1990	5	20.0	8	50	13	31.7
Aug-1990 to APR-1991 Gulf War	2	8.0	3	18.8	5	12.2
MAR-1991 to AUG-2001	6	24.0	2	12.5	8	19.5
SEP-2001 to DEC-2010 OIF/OIF	12	48.0	6	37.5	18	43.9
2011 to present	7	28.0	–	–	7	17.1
Combat Exposure						
Served in Combat Zone	14	56.0	7	43.8	21	51.2

*M* Mean, *SD* Standard Deviation, *OIF* Operation Iraqi Freedom, *OEF* Operation Enduring Freedom

**Table 2:** Baseline Disability Status, Work History, Number of Dependents, and Housing

Variable	IPS (*N* = 25)		Non-IPS-VR (*N* = 16)		Total (*N* = 41)	
	Frequency	Percent	Frequency	Percent	Frequency	Percent
SSI/SSDI						
Yes	5	20.0	8	50.0	13	31.7
No	20	80.0	8	50.0	28	68.3
Filing for SSI/SSDI	3	12.0	1	6.3	4	9.8
VA Service-Connected Disability Status						
None	10	40.0	6	37.5	16	39.0
Filing for the first time	2	8.0	1	6.3	3	7.3
Filing for appeal	–	–	1	6.3	1	2.4
Receiving any SC disability	10	40.0	8	50.0	18	43.9
Filing for increase	3	12.0	–	–	3	7.3
Receiving 100% SC	6	24.0	3	18.8	9	22.0
Employment Status						
Unemployed	21	84.0	14	87.5	35	85.4
Underemployed	4	16.0	2	12.5	6	14.6
Number of Competitive Jobs Held in the Last 3 Years						
Zero	12	48.0	8	50.0	20	48.8
One	4	16.0	5	31.3	9	22.0
Two	6	24.0	–	–	6	14.6
Three or more	3	12.0	3	18.8	6	14.6
Financial Dependents						
Zero	3	12.0	1	6.3	4	9.8
One	10	40.0	9	56.3	19	46.3
Two	5	20.0	5	31.3	10	24.4
Housing						
Single-family Home	6	24.0	4	25.0	10	24.4
Mobile Home	1	4.0	1	6.3	2	4.9
Apartment or Condominium	5	20.0	2	12.5	7	17.1
Renting Room	3	12.0	1	6.2	4	9.8
Transitional housing	3	12.0	3	18.7	6	14.6
Homeless shelter	3	12.0	4	25.0	7	17.1
Motel	1	4.0	–	–	1	2.4
Residential Treatment	3	12.0	1	6.3	4	9.8
Locality						
Urban	19	76.0	13	81.3	32	78.0
Rural	4	16.0	3	18.8	7	17.1
No response	2	8.0	–	–	2	4.9

*SSI* Social Security Income, *SSDI* Social Security Disability Income, *SC* Service-Connected

**Table 3:** Baseline Opioid Use and Treatment History

Variable	IPS (*N* = 25)		Non-IPS-VR (*N* = 16)		Total (*N* = 41)	
	*M*	*SD*	*M*	*SD*	*M*	*SD*
Duration of OUD (years)	10.2	7.9	10.4	12.7	10.3	9.8
	Frequency	Percent	Frequency	Percent	Frequency	Percent
Types of Opioids Used						
Prescription pain pills	19	76.0	11	68.8	30	73.2
Street-derived pills	10	40.0	8	50.0	18	43.9
Street-derived IV, not heroin	2	8.0	1	6.3	3	7.3
Heroin	8	32.0	5	31.3	13	31.7
Current OUD Treatment (past 12 months)						
Any Treatment	21	84.0	10	62.5	31	75.6
Medications for OUD	12	48.0	4	25	16	39.0
Other psychotropic medication	3	12.0	1	6.3	4	9.8
Supportive group therapy	9	36.0	3	18.8	12	29.3
Supportive individual therapy	8	32.0	7	43.8	15	36.6
Complimentary	3	12.0	1	6.3	4	9.8
Inpatient	1	4.0	2	12.5	3	7.3
Residential	5	20.0	5	31.3	10	24.3
Outpatient	2	8.0	2	12.5	4	9.8
OUD Treatment prior to 12 months						
Any Treatment	18	72.0	9	56.3	27	65.8
Medications for OUD	9	36.0	6	37.5	15	36.6
Other psychotropic medication	2	8.0	1	6.3	3	7.3
Supportive group therapy	8	32.0	6	37.5	14	34.1
Supportive individual therapy	8	32.0	8	50.0	16	39.0
Complimentary	1	4.0	3	18.8	4	9.8
Inpatient	6	24.0	4	25.0	10	24.4
Residential	5	20.0	4	25.0	9	22.0
Outpatient	2	8.0	–	–	2	4.8
MINI International Neuropsychiatric Interview						
Major Depressive (current)	3	12.0	1	6.3	4	9.8
Major Depressive (past)	18	72.0	12	75.0	30	73.2
Major Depressive (recurrent)	10	40.0	4	25.0	14	34.1
Agoraphobia (current)	4	16.0	4	25.0	8	19.5
Panic disorder (current)	3	12.0	2	12.5	5	12.2
Panic disorder (lifetime)	8	32.0	5	31.3	13	31.7
Social Anxiety (current)	2	8.0	1	6.3	3	7.3
Obsessive Compulsive (current)	–	–	1	6.3	1	2.4
Posttraumatic Stress (current)	12	48.0	6	37.5	18	43.9
Alcohol use disorder (lifetime)	2	8.0	4	25.0	6	14.6
Alcohol use disorder (past yr)	7	28.0	6	37.5	13	31.7
Substance use disorder (past yr)	17	68.0	10	62.5	27	65.9
Bipolar I (current)	1	4.0	–	–	1	2.4
Bipolar I (past)	–	–	1	6.3	1	2.4
Bipolar II (past)	1	4.0	–	–	1	2.4

*M* Mean, *SD* Standard Deviation, *IPS* Individual Placement and Support, *VR* vocational rehabilitation

### Vignettes

**Vignette #1:** A case randomized to Individual Placement and Support

A veteran enrolled in treatment for OUD 2 years ago after using heroine for about 2 months. He had been treating his pain from lupus with prescribed opioid medication and his attending physician lessened his dosage and he begin using heroine. His provider at the VA referred him to this study when he indicated to provider that he had been living in a hotel room for 8 years and was temporarily living in a basement of a former romantic partner. After screening, the veteran was randomized to IPS services. During the vocational assessment, he communicated a fear of jeopardizing his social security disability income (SSDI) due to employment. The employment specialist drove the veteran to Social Security Services Administration to meet with a representative that informed him of the amount he could make monthly without jeopardizing his benefits. The employment specialist located a job that would enable him to stay under this amount; however, in collaboration with the VA provider a decision was made to wait until the veteran received his new residence so that the veteran would not be faced with too many major life changes at one time. Once the veteran moved into his residence, the employment specialist located another job as a meat cutter (a transitional skill that he developed in the military) at a grocery store. The veteran was offered two jobs as this skill is in high demand. During this time the employment specialist referred the veteran to State Vocational Rehabilitation agency to assist the veteran with securing transportation and clothing for his new position. The Veteran has been employed for six weeks and can work 15 h/week to stay under his income limitation to maintain SSDI. The employment specialist has visited his work location several times and both the veteran and his manager have indicated that he is doing well and enjoying the work.	

**Vignette #2:** A case randomized to non-IPS vocational rehabilitation control arm

Six years ago, the veteran relocated to the area to reunite with his family. He has been unable to secure employment for the past 6 years. The veteran is in recovery from OUD and learned about the study from a family member. He enrolled in the study and was randomized to non-IPS vocational rehabilitation (i.e. treatment as usual) through the VA’s Compensated Work Therapy program. He was placed in transitional work assignment in Environmental Management Services. Although he expressed that he does not particularly enjoy his present position, he is grateful to be making tax-free minimum wage, working 30 h a week. He is hoping to save enough money to start a lawn maintenance business.	

## Discussion

The study protocol outlines a rigorous and comprehensive plan for conducting a two-arm, multi-site RCT to evaluate the efficacy of IPS against a credible active control in a large sample of veterans with history of current or lifetime OUD. The growing prevalence of OUD among military veterans is a critical public health concern, with significant consequences for individuals, their families, and society as a whole. A substantial proportion of veterans with OUD face challenges in achieving stable employment and integrating into community settings. Existing evidence supports the efficacy of IPS in improving employment outcomes for individuals with severe mental illness, PTSD, SUD and veterans in a primary care clinic [8, 9, 12–14]. However, its efficacy among veterans with OUD remains relatively underexplored.

The study's rationale is grounded in the need to address this critical gap in the literature. The potential for IPS to facilitate recovery and reintegration into the workforce among veterans with OUD holds immense significance, not only for the affected individuals but also for the healthcare system and society. A successful IPS program could reduce healthcare costs, enhance overall well-being, and strengthen the economic productivity of veterans [41, 42].

This study is innovative in many ways. First, the use of a mixed-method approach allows the investigators to triangulate results and understand the contextual barriers and facilitators of implementing IPS for veterans with OUD. As mentioned earlier, this study is one of the first to examine the efficacy of IPS in this subpopulation of veterans with OUD. Therefore, understanding these barriers and facilitators of implementation may lead to improved implementation of IPS for other SUDs across the VHA. This promises to close the research-translation gaps that often lead to lags in uptake of effective evidence-based interventions for SUD. Secondly, the use of a CBPR approach allows the integration of multiple stakeholders and partners in informing implementation strategies, thereby leading to greater buy-in. The veteran’s feedback is intentionally and systematically incorporated to ensure a whole health approach to the implementation of IPS among veterans with OUD. Moreover, the goal to examine how employment outcomes inform other psychosocial outcomes is important in our understanding of the mechanisms through which IPS improves community integration. Lastly, to our knowledge and from an extensive review of the literature, our study would be the first to explore the relationship between vocational rehabilitation services, employment and MOUD adherence. This is very important as we know that MOUD adherence is a predictor of positive treatment outcomes among people with OUD [43, 44].

### Implications for practice

The results of this study, if positive, will have far-reaching implications for clinical practice and service delivery in veteran healthcare, and will hopefully lead to better VR services in community-based settings and the general population. A successful IPS intervention would offer a promising and evidence-based approach to improving the vocational outcomes of veterans with OUD. Mental health and addiction treatment providers, as well as VA service organizations, would benefit from guidance on the integration of IPS programs into existing care pathways. It may also foster interagency collaborations to address the complex needs of this population. Incorporating IPS into the continuum of care could empower veterans to achieve stability in their employment, which, in turn, is expected to have a positive impact on other domains of their lives, such as housing stability and overall quality of life [20, 45]. This, in turn, has the potential to reduce the burden on mental health and addiction treatment services, as well as decrease the rates of relapse among veterans [46, 47].

A positive outcome in this study may not only transform the way care is delivered to veterans with OUD but also inform strategies for addressing employment challenges in other populations with SUDs. Furthermore, it underscores the importance of integrated and patient-centered care approaches that consider not only the medical aspects of OUD but also the social determinants that significantly influence an individual's recovery, specifically employment.

This multi-site RCT represents a significant step toward enhancing the evidence base for interventions that can improve the vocational outcomes of veterans with OUD. By addressing these challenges, our research seeks to contribute to the betterment of the lives of those who have served their country and to inform the development of more comprehensive and effective interventions in the field of addiction treatment and vocational rehabilitation.

### Study limitations

The study protocol acknowledges several methodological challenges. Randomized controlled trials offer a robust design for evaluating intervention efficacy, but this approach is not without its limitations. Blinding among participants and study staff is not possible, given the nature of the IPS intervention. However, both IPS and non-IPS VR interventionists have received extensive training to ensure fidelity to intervention arms.

The study also faces potential limitations in terms retention of participants and generalizability. Veterans with OUD are a diverse group, and the study population may not fully represent the entire spectrum of this population. Data collection across multiple sites may introduce variability in intervention fidelity and outcome measurement, which could impact the overall findings.

Over the course of a multi-site trial, it can be challenging to maintain consistent follow-up because of attrition that may lead to missing data, potentially biasing the results. We minimize these potential limitations by keeping assessments to a minimum to reduce the burden on the participant. Finally, the study may be limited by resource constraints, including time and turn-over in personnel which could affect implementation of the intervention and data collection. Given that the sites are within 60 miles of each other and use shared technology, providing temporary coverage for study personnel turnover mitigates these vulnerabilities.

## Conclusions

The intricate relationship between OUD and employment challenges among veterans demands attention from all sectors of society, including health care providers, employers, policymakers, and communities. A holistic approach that addresses the physical, mental, and socioeconomic dimensions of veterans’ lives is essential to break the cycle of OUD and employment struggles. Our study evaluates the efficacy of one such approach, IPS. By recognizing the unique needs of veterans and offering comprehensive support, we can honor their service by helping them reintegrate into the workforce, rebuild their lives, and contribute positively to society. This study responds to the pressing need for evidence-based interventions tailored to the complex and interconnected needs of veterans with OUD. By assessing the impact of IPS on employment, treatment adherence, and psychosocial factors, we aim to not only advance our understanding of the potential benefits of IPS but also to inform the development of more targeted, holistic, and tailored strategies for addressing the multifaceted challenges faced by this vulnerable population.

## Data Availability

The data used in this manuscript can be obtained from the principal investigator of the project upon reasonable request.
